# On the Operation for the Cure of Strabismus

**Published:** 1841-06-19

**Authors:** Joseph Pancoast

**Affiliations:** Professor of Anatomy in the Jefferson Medical College


### ORIGINAL COMMUNICATIONS.

### *On the Operation for the Cure of Strabismus.*

By Joseph Pancoast, M. D., Professor of Anatomy in the Jefferson Medical College.

To the Editors of the Medical Examiner.

*Gentlemen*,—Having, within the last ten months, operated frequently for the cure of strabismus, and a sufficient length of time having elapsed to test the results of the practice, I propose to furnish you, on the present occasion, with some remarks on the subject. I have now operated in all fifty-five times for squinting. In thirty-three cases out of this number, the pupil of the eye was found directed straight inwards, (strabismus convergens;) in fifteen the pupil was directed upwards and inwards, or took this position habitually when the patient was agitated from any cause. In five the squinting was obliquely inwards and downwards, and two were cases of external strabismus. The patients operated on have been of various ages from six to forty, and in one case (a patient of Dr. Moehring of this city) of sixty years of age. This last case was one of double squint, and exhibited a greater amount of deformity than any other that I have met with or seen described. The cornea of the right eye was completely buried in the internal canthus, and had been so during many years. The power of eversion of the ball was almost entirely lost, for, by the utmost efforts of the patient, the lateral margin of the cornea only could be exposed to the light, even when the other eye was closed. Yet this degree of eversion was sufficient to prove that the power of vision in this eye was not destroyed, for, though the rays of light could not enter direct by the pupil, they reached the retina by reflection, from the inner concave surface of the cornea. The objects of vision were consequently not seen in their proper position, but in a direction at right angles with the incident rays, which entered through the outer margin of the cornea. The eye ball was protruded unusually far between the lids, in consequence of the excessive action of the oblique muscles: the insertion of the inferior oblique from the inverted state of the ball being plainly visible near the outer angle of the lids, showing the muscle to be in a tense condition. The left eye was amaurotic, and squinted inwards, and could not be turned out by the patient so as to be made quite straight in the orbit.

This case, which was successfully treated by operation, in the presence of Dr. Moehring, and Dr. Folts of New Hampshire, I more particularly notice in these brief remarks, from the extraordinary degree of deformity which existed, as it seems to show that though it be more easy to correct the defect earlier in life, there is scarcely any instance in which relief may not be obtained, if the operation be properly apportioned to the peculiar exigencies of the case. It was attended besides, as has been usually observed in such cases, with most manifest improvement in the power of vision.

In an operation which is usually so simple as that for the cure of strabismus, attended with scarcely any pain, and often with the effusion of but a few drops of blood, and which any surgeon is capable of performing, there is, notwithstanding, much judgment required in suiting the operation to the different varieties of strabismus, so as to ensure a permanently successful result. A simple division of the tendon of the muscle at fault, as first recommended, will rarely ever, I believe be found to answer the purposes intended. But in a very great majority of cases, the section of the tendon, and a free division of the insensitive submuscular fascia below it, at its insertion upon the ball, will be found fully to suffice.

Cases, however, will be frequently met with where the cornea has been for a long time habitually drawn inwards and upwards, or inwards and downwards, or straight inwards, and at the same time slightly sunk in the orbit, where we have the superior or inferior rectus, as the case may be, concerned with the internal rectus in producing the deformity. In such instances, beside the section of the internal rectus and its fascia, one of the other two muscles must be in part or wholly divided before the eye can take its straight position in the orbit. By a careful observation of the movements of the ball previous to the operation, or after the section of the internal rectus, it may readily be determined which one of the other two is to be subjected to the action of the scissors. The stays having thus been divided on the internal surface of the ball, the oblique muscles will protrude the eye, so as to remove its sunken appearance. Occasionally they give it an unusual degree of prominence, which does not, however, constitute a permanent deformity, as I have in every instance seen it to disappear when the divided muscle or muscles had formed a re-attachment to the ball, and resumed their usual offices.

The greatest care is required, however, not to carry the operation too far, by dividing more than is necessary, and especially if we operate upon both eyes at the same sitting, in cases of double squint. The golden rule to be observed is not to cut a single fibre more of muscle or fascia than is necessary to put the eye straight, and take away from the patient, for a few days, the power of turning it again in the vicious direction. But the operator should be careful to put it completely into the straight position before he leaves it, for experience has shown to me that little reliance is to be placed upon the action of the other muscles overcoming any remaining deformity. Immediately adjoining the sclerotic coat will be found a deep-seated fascia beneath which run some bundles of vessels to supply the iris. This fascia should never be opened, as it exposes to the wounding of these vessels, from which the profuse hæmorrhage that some have met with in the operation has no doubt proceeded.

When squinting has been long habitual, it is rarely limited to a single eye. Both will very often be found involved, though usually in different degrees. This is frequently the case even without the knowledge of the patient, for, from his habit of attending to the impressions of a single eye, that one in which the sight is most perfect will be found usually straight in the orbit, while the whole amount of squint common to both will be collected in the inner angle of the other. The squint, in what was thought the unaffected eye, only becoming apparent to the patient after the deformity had been corrected by operation on the other. It becomes, therefore, very desirable to possess a rule of judging of the exact degree of strabismus existing in each eye previous to operation. A very simple plan, which I have usually found sufficient to determine the point, is to cause the patient to look to the extreme right and left alternately, and the extent of the white that he cannot hide by the eversion (and which, in the sound eye, can always be hid) marks the degree of obliquity in each respective eye, in cases of internal squinting. In some instances, and especially among children, where the patient possesses an unusual power of rotation over the ball, or even in the adult, where the opening between the canthi is unusually narrow, or where the eyeball is much sunken, the white may be all hid by eversion even where the strabismus exists to some extent. In these cases of exception the rule cannot be applied with the same degree of precision; it becomes necessary, then, to watch the relative degrees in which the pupil can be turned outwards.

In the operation for strabismus, the only really painful part of the process, provided much poking with the blunt hook be avoided, is the forcible separation of the lids by the different mechanical contrivances, such as the various specula and levers, that have been employed. I am, therefore, in the habit now, when the patient is docile, and feels the importance of the operation, of having the lids simply separated with the fingers of an assistant, which plan sufficiently exposes the ball, and is far less trying to the patient, as the lids can be instantly raised or lowered, if it be necessary, as every judicious surgeon will find it at times, to pause for a few moments and watch the effect of his operation. It also expedites the process, for the cutting part of the operation may be very quickly done, while the adjustment of the complicated specula necessarily requires some time. When mechanical means are required for the separation of the lids, I find, after trying most of the kinds that have been suggested, none to answer better than the simple elevator of Pellier for the upper lid, while the lower can in all cases be readily depressed by the finger of an assistant.

The only instruments which I find it necessary to use, and which I think will suffice for any one who is ambidextrous, is a small hook with a bifid or trifid point, to hitch into the tunica albuginea, and turn the eye-ball moderately outwards, (if it be a case of internal strabismus,) and a pair of sharp-pointed angular scissors, to separate the plica semilunaris from the ball, and divide the tendon and fasciæ below it. A blunt hook should also be at hand, as it will be found occasionally useful when it is necessary to raise up any deep seated bands of contracted fascia, in order to free the ball, or to draw out and divide, in those rare cases that demand it, the tendons of the superior or inferior rectus. If the left eye be operated on the hook must be held in the right hand, and the scissors in the left; the instruments must be reversed in the hands if the right be the subject of operation. By this mode of proceeding, the surgeon is able to judge better of the effect of his incisions, by feeling himself the resistance to diminish to the hand that holds the hook, than when an assistant is employed with an extra instrument, to hold the eye outwards.

In regard to the action of the oblique muscles, and the necessity of dividing them for the cure of squinting, much contradictory testimony has been furnished by different writers. From carefully noticing their action when drawn upon in the dissected organ, from an experimental division of them in the eye of the dog, and from watching the effect of operations upon them in the human subject, I am satisfied they are rarely, if ever, concerned in producing strabismus.

The only cases in which they should be divided, is where, by their undue action, they have produced the protruded and staring position of the ball that is sometimes met with. To remedy this defect, I have divided the inferior oblique muscle twice, once near its origin, and once at its insertion upon the ball—and the superior oblique three times. The division of the latter muscle appears to have the most effect in allowing the recti muscles gradually to replace the eye-ball at its proper depth in the orbit. Except in the case of protrusion described, the section of these muscles is wholly uncalled for, and would be, so far as the motions of the eye are concerned, without any obvious effect. In regard to the action of the inferior oblique muscle, I believe there is much misconception—and there are some facts in regard to its anatomy, which have escaped observation, that it is my intention hereafter to point out. In the case of the patient of Dr. M., alluded to in the early part of this paper, the first step of the operation was, to divide the inferior oblique at its insertion, and which had no effect whatever in diminishing the force of the fixed inversion of the ball. By the ordinary operation I practise, described above, the eye was made straight. But the inferior oblique did not again form its connection with the ball. Ten days after the operation, on causing the patient to make an effort to turn the eye in, the inferior oblique could be seen twitching the cellular tissue and conjunctiva, to which it had become attached at the outer margin of the lower lid—a movement of a similar description being observable in the external rectus. Here it was evidently associated with the action of eversion. What is the peculiar office of that muscle, I shall not, at present, attempt to explain. It certainly was not one of the causes which produced the fixed state of inversion of the eye. Experience in this case, and every other where it has been cut, is against such a conclusion.

The operation for strabismus, if properly practised, and in suitable cases, will, I believe, be found uniformly successful. In none of my cases has it been followed by pain, or inflammation, calculated to give any serious uneasiness. In four only, out of the fifty-five operated on, has the operation failed to restore the eye completely and permanently to its natural position. In two, the eyes, which were amaurotic, but greatly distorted by inward squint, have gone a very little too far outwards. And in the two other cases, which were among the first I operated on, another operation will complete the cure, the deformity being in a great measure removed by the first.

Where we operate on a single eye, and the retina is not insensitive, there is no danger, unless there has been a wanton use of the scissors, or the operator lacks experience, of the eye turning too far in the opposite direction. If it should pass a little beyond the middle line at first, it will in a few days become straight. There is a self-adjusting power in the eye itself, by which the remaining undivided muscles are involuntarily brought into action to keep the sensitive centre of the retina presented forwards, to the rays of light. This appears to me as the great safeguard against the production of deformity after the operation; and the eye operated on, should not, therefore, after the first few hours have passed, be excluded from the light. But when the retina of one eye is insensitive, as in amaurosis, and we are called on to operate for the removal of the deformity, and with the hope of bettering the vision which is frequently realized, this self-adjusting power does not exist, and there is some risk of producing distortion of another kind, if we do not divide with less freedom than usual the bands that hold the eye in. In the operation for double squint, in both eyes at the same sitting, a plan which has some advantages to recommend it, a cautious division of the tendon and fascia of the eye least affected, is also, I believe, highly necessary; for the external recti seem each to me to act by association with more power, when both the internal recti are divided, than when one only is cut.

I have believed, gentlemen, that these remarks, in reference to a new and beautiful, but simple and successful operation, might be found of some interest, as they have been derived from experience; and few, I believe, as yet, have had the opportunity, in this country, of operating more frequently than myself.

